# Factors associated with stillbirth in selected countries of South Asia: A systematic review of observational studies

**DOI:** 10.1371/journal.pone.0238938

**Published:** 2020-09-16

**Authors:** Samikshya Poudel, Pramesh Raj Ghimire, Nawaraj Upadhaya, Lal Rawal

**Affiliations:** 1 Ujyalo Nepal, Ratnanagar Municipality, Chitwan, Nepal; 2 Department of Research and Development, HealthWorks, Amsterdam, The Netherlands; 3 School of Health Medical and Applied Sciences, CQUniversity, Sydney, Australia; 4 Translational Health Research Institute (THRI), Western Sydney University, Sydney, Australia; La Trobe University, AUSTRALIA

## Abstract

**Background:**

Despite having the high rate of stillbirth in most of the countries of South Asia, there is a lack of synthesized evidence based on factors associated with stillbirth. This study systematically synthesizes the evidence on factors associated with stillbirth in the four selected countries of South Asia.

**Methods:**

This review was conducted using Preferred Reporting Items for Systematic reviews and Meta-Analyses (PRISMA) guidelines. Studies that examined factors associated with stillbirth in South Asia were searched using five major electronic search databases including MEDLINE, CINAHL, Embase, PsycINFO, and Scopus, published between January 2000 and December 2019. In the meta-analysis, significant heterogeneity was detected among studies (I^2^ >50%), and hence a random effect model was used.

**Results:**

A total of 20 studies met the inclusion criteria. The pooled rate of stillbirth from the studies in Bangladesh, India, Nepal, and Pakistan was 25.15 per 1000 births. Pregnancy complications, maternal health conditions, fetal complications, lack of antenatal care, and lower Socio Economic Status (SES) were the most common factors associated with stillbirth in countries of South Asia.

**Conclusion:**

This study confirmed that stillbirth in selected countries of South Asia remains high. To reduce stillbirth, a greater focus needs to be on timely management of preterm labor, maternal hypertension, and provision of financial support for quality antenatal and delivery care. The interventions should be targeted for women living in remote areas, who are less educated and those with low SES.

## Introduction

For a global comparison, stillbirth (antepartum and intrapartum) is defined as a death of a fetus ≥ 28 weeks of gestation [1]. Annually, stillbirth is estimated to occur in 2.6 million pregnancies worldwide [2, 3]; and of these, 98% are reported from low-middle income countries with prematurity, intrapartum complications and infections being the leading causes of these untimely deaths [4, 5]. About 1.4 million stillbirths occur during labor and birth [4] which could be easily prevented; but due to poor infrastructure, lack of qualified health care providers, and poor quality of antenatal, delivery and postnatal care, this has not been possible in low resource settings [6, 7]. In most developing countries, in the absence of cost-recovery mechanisms, stillbirth is a significant health and economic loss, largely due to direct healthcare costs incurred during medical investigations; long-term mental health impacts on grieving parents; and adverse health consequences on surviving siblings [8–11]. In order to address a wide-range of negative health outcomes and economic consequences, the 67th World Health Assembly in 2014 endorsed the World Health Organisation (WHO) Global Action Plan for preventing stillbirth [1]; and stillbirth has become a visible maternal and child health agenda in the era of Sustainable Development Goal (SDG) [12]. To prevent stillbirth, this Global Action plan has recommended cost effective intervention packages in the continuum of care from antenatal identification and subsequent management of pregnancy complications to skilled birth attendants [1]. In addition, this action plan has emphasized the need for high quality research to prioritize intervention packages [1]. Despite these global developments, stillbirth remains a neglected issue, does not feature explicitly in policies and programs and is under-financed [3, 13].

Asia is home for over half of the estimated global stillborn babies; and the burden of stillbirth is inequitably distributed across Asia: the reported rate is as high as 26 per 1000 births in South Asia, and as low as 7 per 1000 births in East Asia [4]. Hence, to capitalize global and regional Every New-born Action Plan targets ≤12 stillbirths per 1000 births by the year 2030 [1], it is imperative to close this gap that requires prioritizing WHO recommended cost-effective intervention for populations with higher risk of stillbirth. It has been suggested that identification of intervention priorities from individual published studies is problematic [14]; and hence, a systematic synthesis of evidence-based published studies across countries of South Asia would contribute to identify priority population for effective regional interventions.

A systematic review on factors associated with stillbirth in low and middle income countries published in 2013 by Aminu and colleagues [15] included 4 out of 8 South Asian countries and provides valuable information on factors associated with stillbirth. However, since 2013 there have not been any reviews in this topic, despite substantial public health research carried out to examine factors associated with stillbirth across South Asia. Hence, this study aimed to synthesize the latest literature on stillbirth, conduct comparative analysis of rates and factors associated with stillbirth across South Asian region, and provide an updated evidence-base in key areas that require special attention for public health interventions.

## Methods

### Outcome measure

The primary outcome measure of this study was stillbirth, defined as a death of a fetus ≥ 28 weeks of gestation [1].

### Search strategy

The reporting of this study was conducted according to the Preferred Reporting Items for Systematic reviews and Meta- Analyses (PRISMA) guidelines [16]. Literature search was performed using five electronic databases (MEDLINE, Embase, CINAHL, PsycINFO, and Scopus). The lead author (SP) conducted the initial database search on 2 January 2020. Keywords and relevant MeSH headings were used to identify records for each of the three main concepts (stillbirth, risk factors, and South Asian countries); and the records obtained for each key concepts were combined using Boolean operators as shown in S1 Table.

All the records retrieved from the five databases were then imported into an EndNote library for screening. A detailed search strategy used in MEDLINE has been presented in S1 Table.

### Inclusion and exclusion criteria

The eligibility of retrieved studies was assessed based on a set of five inclusion and exclusion criteria. Studies were included if they were based on stillbirth and assessed at least one of the factors associated with stillbirth; were conducted in countries in South Asia (Nepal, Bhutan, India, Maldives, Bangladesh, Sri lanka, Pakistan, Afghanistan); were written in English language; were published in peer-reviewed journals; and were published between January 2000 and December 2019. The reason of choosing January 2000 is to understand factors associated with stillbirth after the endorsement of millennium development goals so that the findings from this study would shed light for the implementation of sustainable development goals strategy to achieve Every Newborn Action Plan target of 12 or fewer stillbirth per 1000 births by the year 2030. The inclusion and exclusion criteria as well as data extraction categories were further discussed and decided in consultation with other authors (PG and NU).

### Data extraction

During the data extraction process, the first author (SP) initially imported all the identified articles into an EndNote library and removed the duplicate records. SP then screened the records based on reading titles and abstracts of the retrieved articles. In the final screening phase, full texts of retrieved articles were identified using various electronic search engines (electronic databases, google, google scholar). While applying inclusion and exclusion criteria, articles that met inclusion criteria were retained for this review. Data extraction and appraisals of each retained study were independently reviewed by SP and PRG; and any disagreements between the two reviewers were resolved through consensus and further consultation with other authors (NU and LR). To retrieve all the relevant articles, we performed a search of the bibliographical references of all articles that met inclusion criteria followed by citation tracking with Google Scholar.

### Data analysis

The rate of stillbirth was extracted from each of the 18 observational studies included in this review. Two studies were excluded in the meta-analysis because of lack of information to compute stillbirth rate [17, 18]. The syntax “metaprop” in Stata version 14.0 (StataCorp, College Station, TX, USA) was used to generate forest plots. Each forest plot showed the rate of stillbirth reported in 18 reviewed studies and their corresponding weights, as well as the pooled rate across studies and its corresponding 95% Confidence Intervals (Cl). A test of heterogeneity of the data obtained for the different studies showed a high level of inconsistency (I^2^ > 50%) across studies, thereby warranting the use of a random effect model in the meta-analysis.

## Results

**Fig 1:**
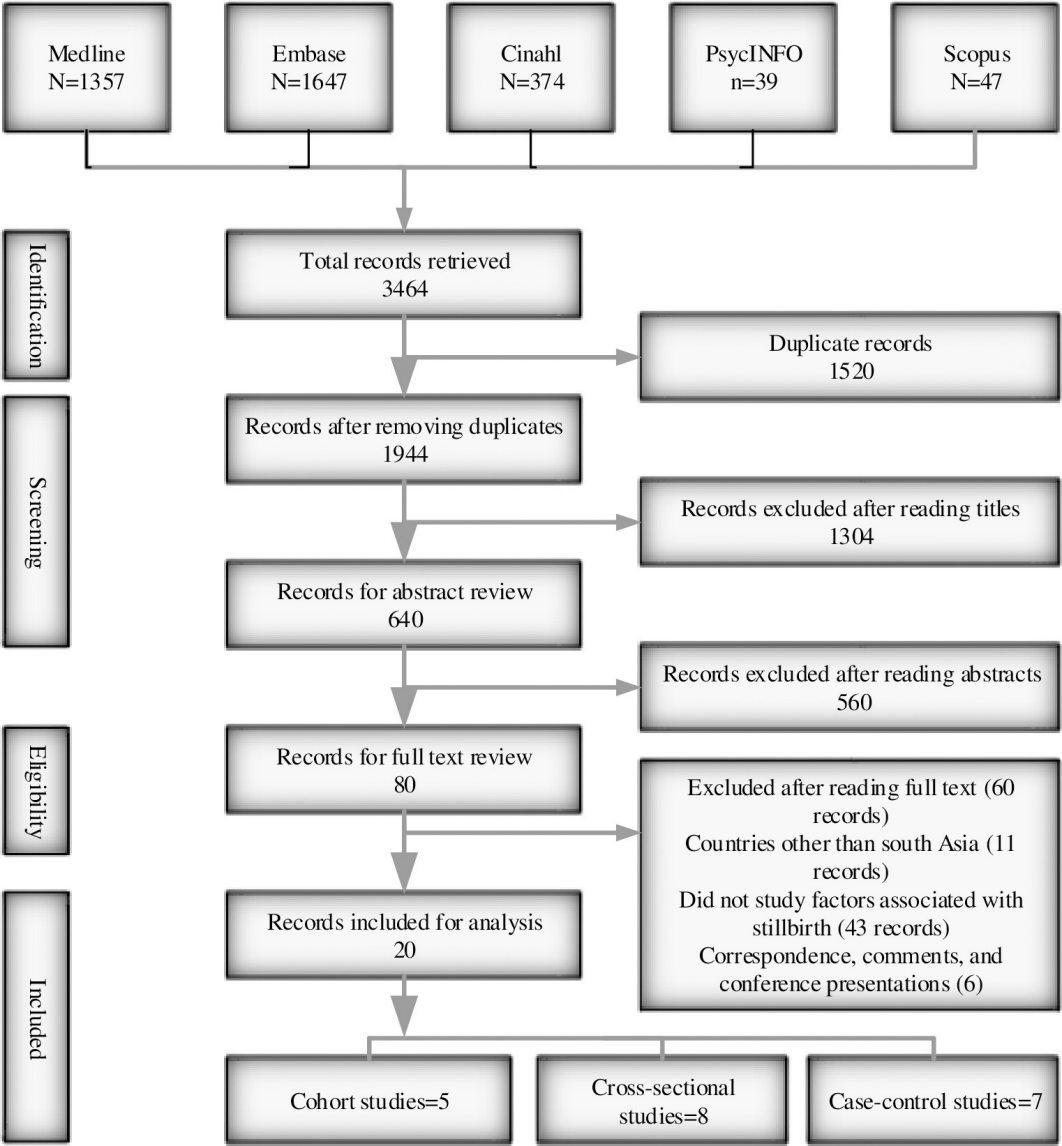
Inclusion and exclusion of studies for review.

After applying inclusion and exclusion criteria on 3464 records, a total of 20 studies met the inclusion criteria for this review (Fig 1).

### Characteristics of reviewed studies

Of the 20 studies, five were cohort [19–23], eight were cross-sectional [24–31], and seven were case-control studies [17, 18, 32–36] (S2 Table). Seven studies were conducted in Bangladesh; six studies were conducted in India; three studies were conducted in Nepal; and four studies were conducted in Pakistan. This review did not find studies from Afghanistan, Bhutan, Maldives and Sri Lanka. The sample population in selected studies ranged from 362 to 1,88,917.

### Stillbirth rate

**Fig 2:**
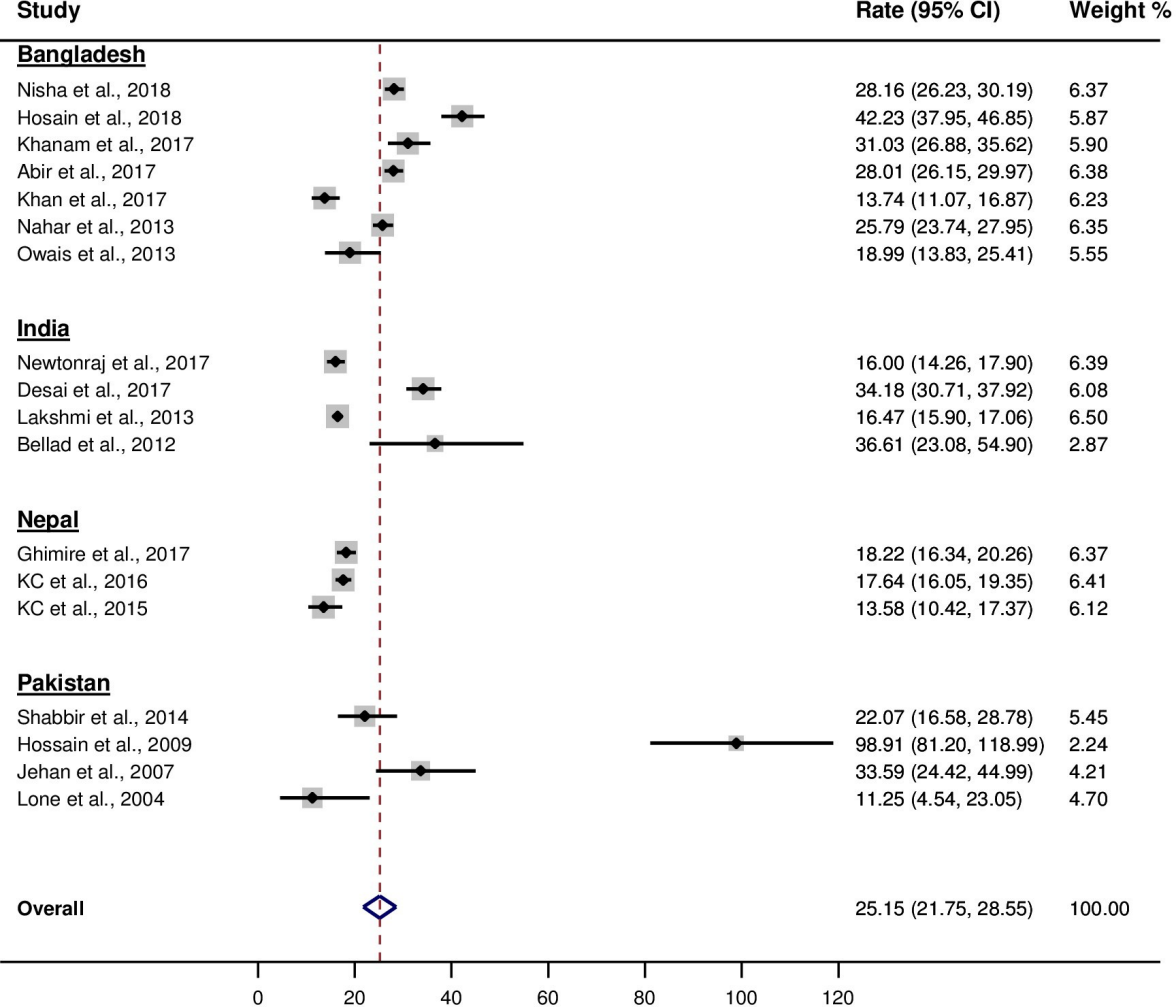
Stillbirth rate per 1000 births obtained from reviewed studies in South Asia.

The pooled stillbirth rate obtained from 18 reviewed studies was 25.15 [95% CI: 21.75, 28.55] per 1000 births (Fig 2).

### Factors associated with stillbirth

Six studies examined the association between prematurity and stillbirth; of which, four case control studies conducted in India [17, 35], Bangladesh [36], and Nepal [33]; and two cross-sectional studies conducted in India [26], and Pakistan [29] found a significant association between premature labour and stillbirth (Table 1). Maternal hypertensive disorder during pregnancy was associated with stillbirth in India [17, 18], Pakistan [29], Nepal [32], and Bangladesh [25].

**Table 1:** Factors associated with stillbirth obtained from reviewed studies by selected countries in South Asia.

**Bangladesh**	**OR/RR**	**95% CI**	**Nepal**	**OR/RR**	**95% CI**
Polluting fuels (Ref: Clean fuels)	1.25	(0.85, 1.84)	Maternal age≥25years (Ref: <18 years)	1.77	(1.12, 2.82)
Maternal age (20–29 years) (Ref: 19 years or below)	0.64	(0.53, 0.78)	Mothers who lived in mountains or hills (Ref: Terai)	1.71	(1.1, 2.66)
Higher birth order: 2+ children (Ref: 1)	2.10	(1.73, 2.54)	Mothers whose religion was Hindu, Muslim, Christian and others (Ref: Buddhist)	1.38	(1.02, 1.87)
Higher birth order: 3+ children (Ref: 1)	4.57	(3.52, 5.92)	Mothers who had no schooling or primary level of education (Ref: Secondary or higher)	1.72	(1.1, 2.97)
Daily smokeless tobacco consumption >5 times (Ref: Less frequent users)	5.89	(1.70, 20.30)	Mothers whose major occupation is agriculture (Ref: Not working)	1.80	(1.16, 2.78)
Pregnancy induced hypertension	1.80	(1.30, 2.50)	Open defecation (Ref: Improved sanitation)	1.48	(1.00, 2.18)
Maternal education (Secondary and higher) (Ref: No schooling)	0.59	(0.43, 0.82)	Father with no schooling (Ref: Secondary or higher)	1.71	(1.10, 2.64)
Maternal education (Primary) (Ref: No schooling)	0.66	(0.55, 0.80)	Poor wealth quintile (Ref: Non-poor)	1.80	(1.10, 3.40)
Mothers with more than 2 children (Ref: 1 child)	0.56	(0.46, 0.69)	Lower maternal education (Ref: Secondary or more)	3.20	(1.80, 5.50)
Mothers with more than 3 children (Ref: 1 child)	0.49	(0.39, 0.63)	Lack of antenatal care visit (Ref: At least 1 visit)	4.80	(3.20, 7.20)
Mothers with more than 4 children (Ref: 1 child)	0.53	(0.43, 0.66)	Antenatal haemorrhage	2.10	(1.10, 4.20)
Poor household (Ref: Rich household)	1.47	(1.13, 1.90)	Multiple birth	3.00	(1.90, 5.40)
Mothers who had no access to newspaper (Ref: Access to newspaper)	1.34	(1.02, 1.76)	Obstetric complication during labour	4.50	(2.90, 6.90)
Mothers with 3 or more under five children (Ref: 1–2 under five children)	0.70	(0.63, 0.88)	Lack of fetal heart rate monitoring per protocol	1.90	(1.50, 2.40)
Obesity (Ref: Normal weight)	3.20	(0.77, 13.55)	Lack of partogram use	2.10	(1.10, 4.10)
Maternal age≥35 years (Ref: 20–35 years)	2.90	(1.50, 25.50)	Preterm birth	5.40	(3.50, 8.20)
Maternal illiteracy (Ref: Post primary)	1.60	(1.10, 2.20)	Preterm birth with small for gestational age	9.00	(7.30, 15.50)
Premature delivery (<37 weeks)	5.20	(3.20, 8.50)	Increasing maternal age	1.00	(1.00, 1.10)
Prolonged labour	2.80	(1.60, 4.60)	Less than 5 years of maternal education (Ref: Six years of education or more)	2.40	(1.70, 3.20)
Decreased fetal movement	25.50	(5.70, 113.20)	Increasing parity (Ref: Primi)	1.20	(1.00, 1.30)
Fetal distress	7.30	(1.30, 42.40)	Previous stillbirth	2.60	(1.60, 4.40)
Failure to progress in labour	2.40	(1.10, 5.50)	No antenatal care attendance (Ref: At least one attendance)	4.20	(3.20, 5.40)
History of bleeding during pregnancy	22.40	(2.50, 197.50)	Maternal hypertensive disorder during pregnancy	2.10	(1.50, 3.10)
**India**			Small for gestational age babies (Ref: Appropriate for gestational age)	1.50	(1.20, 2.00)
<10 minutes time (after the onset of labor) to attend initiate the management of delivery	3.60	(2.50, 5.10)	**Pakistan**		
Untreated hypertension during pregnancy	2.90	(1.50, 5.60)	Grand multiparity	1.16	(1.05, 1.30)
Presence of any complication during labour, warranting treatment	1.70	(1.20, 2.40)	Obstructed labour	16.20	(5.50, 47.70)
older age of mother (increasing age)	1.10	(1.00, 1.20)	Cord prolapse	7.30	(2.20, 24.20)
Vaginal delivery	8.10	(2.60, 26.00)	Hypertensive disorder	9.60	(4.00, 23.00)
Induced labour	2.60	(1.50, 4.50)	Abruptio placenta	137.00	(52.7, 356.30)
Green or light brown liquor	2.00	(1.10, 3.80)	Placentae previa	71.20	(21.9, 230.70)
**India**	**OR/RR**	**95% CI**	**Pakistan**	**OR/RR**	**95% CI**
Preterm delivery	6.40	(3.70, 11.00)	Preterm labour	15.60	(4.50, 54.20)
Smaller number of household member	1.20	(1.10, 1.30)	Gestational diabetes	31.10	(8.20, 118.9.0)
Sickle cell disease	2.43	(1.31, 4.53)	Ruptured uterus	49.80	(11.80, 211.00)
Preterm delivery	3.50	(2.10, 6.00)	Fetal distress	13.10	(5.00, 34.50)
Previous history of stillbirth	4.00	(2.10, 7.80)	Low gestational age of the baby	0.77	(0.70, 0.90)
History of intake of SSD	2.60	(1.50, 4.50)	Weight of the baby	0.65	(0.40, 1.00)
Complication during labour	3.30	(2.10, 5.30)	Foul smelling amniotic fluid	4.60	(2.10, 9.80)
Presence of high blood pressure during pregnancy	1.80	(1.04, 3.10)	Hemoglobin level<8gm/dl	3.80	(1.60, 9.20)
Biomass fuels for cooking at home (Ref: LPG/electricity)	1.24	(1.08, 1.41)	Cloudy or meconium stained fluid	12.10	(5.60, 25.80)
Illiterate mother	1.13	(1.04, 1.23)	Excessive bleeding during delivery	5.50	(2.70, 11.20)
Illiterate father	1.14	(1.06, 1.23)	Maternal anaemia	3.70	(0.86, 14.60)
Primigravida (Ref: 2 or 3)	3.49	(3.18, 3.82)			
Multigravida (Ref: 2 or 3)	0.53	(0.48, 0.58)			
History of previous abortion	37.84	(34.13, 41.98)			
Unskilled antenatal care (Ref: Skilled antenatal care)	2.08	(1.43, 3.05)			
Age at last pregnancy≥35 years (Ref: 20–34 years)	1.45	(1.26, 1.65)			
Bleeding complications	1.57	(1.44, 1.71)			
Fetal complications	1.90	(1.73, 2.09)			
Other complications	1.13	(1.03, 1.24)			
Prematurity	1.56	(1.44, 1.69)			
Home delivery	0.75	(0.68, 0.82)			
Consanguineous marriage	2.47	(1.00, 6.10)			

CI: Confidence Interval; OR: Odd Ratio; RR: Relative Risk; SSD: Sex Selection Drug; Ref: Reference.

Complications such as antepartum haemorrhage in Bangladesh [20, 25], India [26], and Nepal [33]; and intrapartum haemorrhage in Bangladesh [22], and India [26] were also found to be associated with stillbirth. Maternal anaemia was reported to be associated with stillbirth in two studies conducted in Pakistan [21, 22]. A study conducted in India also found a significant association between the sickle cell disease and stillbirth.[30]. Stillbirth was also associated with prolonged labour or failure to progress in labour in Bangladesh [36], and induced labour and present of green or light brown liquor discharge during pregnancy in Chandigarh, India [35]. Complications during labor and its association have been documented in several studies [17, 18, 26, 29]. For example, convulsions, excessive bleeding, fever, high or low blood pressure, obstructed labor, excessive perspiration, blurring vision, and kidney failure in India [26]; and obstructed labor, abruptio placentae, placenta previa, cord prolapse, gestational diabetes, ruptured uterus in Pakistan [29] were also found to be associated with higher stillbirth. Labor and delivery characteristics including foul- smelling amniotic fluid, cloudy or meconium-stained fluid were identified as associated factors for stillbirth in Pakistan [22]. Prolonged labor and maternal fever were not found to be significantly associated with stillbirth in Pakistan [22]. Grand multiparity mothers with ≥4 children were more likely to have stillbirth in India [26], Nepal [32], and Pakistan [29]. However, a cross sectional study conducted in Bangladesh[28] found that mothers with more than two children were less likely to have stillbirths compared to mothers with one child. Obese women were at three times higher risk of stillbirths than normal weight women in Bangladesh [24].

Several fetal complications such as fetal distress, decreased fetal movement, small for gestational age and under weight babies were investigated and found to be associated with stillbirth in Nepal [32, 33], Pakistan [29], India [26], and Bangladesh [36]. Similarly, Previous history of stillbirth was found to be a risk of stillbirth in Nepal [32] and India [17]. A study conducted in Nepal also found that compared to singleton, multiple birth was more likely to stillborn [33].

Lack of antenatal care were found to be associated with the risk of stillbirth in two studies conducted in Nepal [32, 33] and a study conducted in India [26]. Lakshmi et al, also found that mothers giving birth at home, and those who used biomass energy for cooking at home were more likely to have stillbirth in India [26]. Similarly, a study conducted in Bangladesh [31] found that women who were exposed to polluting fuel were more likely to have stillbirth. In addition, this Bangladeshi study also revealed that women having higher birth order (>2) were significantly at higher risk of stillbirth.

A cross-sectional study conducted in India [26], three case control studies conducted in Bangladesh [36], India [35], and Nepal [32]; and a cohort study conducted in Pakistan [19] reported that women with advanced maternal age (≥ 35 years) were more susceptible to stillbirth. In addition, a study from Nepal also found that maternal age at first birth (≥ 25 years) to be associated with increased stillbirth [27]. Contrary to a Nepalese study [27], a cross-sectional study conducted in Bangladesh found that younger maternal age (<20 years) was significantly associated with stillbirth. The impact of maternal education on stillbirth was examined by six studies conducted in Nepal [27, 32, 33], India [26], and Bangladesh [28, 36] which found that lower maternal education was significantly associated with the higher risk of stillbirth. Mothers who did not read the newspaper [28], whose religion was Hindu, Muslim, Christian and others [27], smaller number of household [35], husbands occupation as unskilled labor [36], and consanguineous marriage [23] are several sociodemographic factors found to be associated with stillbirth in Bangladesh, India, and Nepal [23, 27, 28, 35, 36]. Ghimire et al. found that mothers who lived in mountains or hills were at higher risk of stillbirth compared to their Terai counterparts [27]; and a study conducted in India found that more than 10 minutes time (after the onset of labor) to initiate the management of delivery was also linked to higher risk of stillbirth [18]. In low-middle income countries, due to weak health care management, it takes longer time to attend delivery. The longer waiting time at the health care centre after the onset of labor, especially for complicated delivery is a risk for stillbirth as in those countries women would have already taken longer time to arrive to the health facility.

## Discussion

This systematic review provides a recent evidence-base on stillbirth rate and factors associated with stillbirth in countries of South Asia. The stillbirth rate in South Asia currently stands quite high and the countries in the region are unlikely to achieve the Sustainable Development Goal target of 12 stillbirths per 1000 birth by 2030. Our findings suggest that the most common factors associated with stillbirth in countries of South Asia include preterm labour, maternal hypertensive disorder, advanced maternal age, poor household wealth status, lower or no educational status, and lack of antenatal care. In these South Asian countries, policies around improving maternal and child health outcomes do exist [37–40] but the main problem is poor implementation of these policies and strategies, which partly due to inadequate health financing and weak health care systems in these countries.

Maternal health conditions were determining factors for stillbirth in South Asia. For example, complications during pregnancy such as hypertension, pre-eclampsia, ante/ intra partum haemorrhage, gestational diabetes, and anaemia were identified as major factors for stillbirth. These findings coincide with studies conducted in China [41], Indonesia [42], and Ethiopia [43]. Consistent with a study in our review [24], a cross-sectional study conducted in Nigeria [44] also found an increased risk of stillbirth among mothers who were obese. Quality antenatal care helps to monitor and improve maternal and fetal health during the antenatal period; and the higher risk of stillbirth among mothers who did not receive antenatal care has been clearly established in studies from India [26] and Nepal [32, 33].

Several socio-demographic variables were found to be the determining factors for stillbirth in South Asia. The association between lower socioeconomic status and higher stillbirth in this review is in agreement with previous studies conducted in other developing countries [44, 45]. In addition, this review found a significant association between stillbirth and lower maternal education in Nepal, India, and Bangladesh; consistent with the Demographic and Health Survey in the African Great Lakes region [46]. However, studies conducted in Pakistan have found no significant association between stillbirth and maternal education [22, 29]. Advanced maternal age is a known risk factor for stillbirth; and the association between advanced maternal age and stillbirth has been clearly established in studies conducted in developed and developing countries [14, 46]. This review also found an advanced maternal age to be an important risk factor for stillbirth in South Asia. It is important to note that maternal age has been defined inconsistently across studies included in this review[26, 27, 32, 35, 36]; and contrary to a Nepalese study [27], a similar study [31] that used data of Bangladesh Demographic and Health Survey found a protective association between younger maternal age (20–29 years) and stillbirth.

In this study, a lack of fetal movement and fetal complications were found to be associated with stillbirth. Similar findings have been reported in a systematic review conducted in developing countries where congenital anomalies accounted for 2.1–33.3% of stillbirths, placental causes (7.4–42%), asphyxia and birth trauma (3.1–25%), umbilical problems (2.9–33.3%), and amniotic and uterine factors (6.5–10.7%) [15].

In this review, the choice of predictor variables used to examine factors associated with stillbirth across studies vary substantially. Notably, studies that used secondary data of Demographic and Health Survey from Nepal and Bangladesh [27, 28, 31] have primarily examined distal factors associated with stillbirth; whereas the remaining studies in this review have examined proximal determinants including maternal health condition, fetal complication, maturity and perinatal care. The reason for non-use of proximal determinants in studies that used Demographic and Health Survey has been described by Christou et al [47].

The present review has strengths and limitations. This is a first comprehensive search for evidence on factors associated with stillbirth in South Asia. The methodological approach used in this systematic review also includes an extensive search of five electronic databases with validated search strategies. However, this review is limited by number of factors. First, the prescribed methods of this systematic review do not allow for comprehensive coverage. For example, relevant studies published in a language other than English might have been missed out. A second limitation of this review is that due to heterogeneity among studies in terms of definition used for variables examined, study design, sampling representativeness, and statistical measures applied, a formal meta-analysis was not conducted. Third, stillbirth can be viewed as antepartum or intrapartum; and majority of the studies included in this review do not distinguish whether the death was antepartum or intrapartum. Therefore, the rate and factors associated with stillbirth in this study should be used with caution while formulating intervention that are specific to timing of stillbirth.

## Conclusion and policy implications

This study confirmed that stillbirth in selected countries of South Asia remains high. Our findings suggest that preterm labour, maternal hypertensive disorder, advanced maternal age, poor household wealth status, lower or no educational status, and lack of antenatal care were associated with stillbirth these countries. In order to address the problem of stillbirth and achieve maternal, neonatal and child health outcomes, there is a need for developing and implementation of effective interventions that minimize the modifiable risk factors of stillbirth, such as improving health and nutritional status of pregnant women, antenatal care, healthy behaviour during pregnancy, control overweight/ obesity and smoking cessation. The interventions need greater focus on timely management of preterm labor, maternal hypertension, and provision of financial support for quality antenatal and delivery care and should be targeted for women living in remote areas, who are less educated and those with low SES. Further, the quality of maternal and child health care services should be improved with assurance of access to and availability of trained health care providers, quality equipment and medication and emergency neonatal care services at health facilities around the clock.

## Supporting information

S1 Table Detailed search strategy used in MEDLINE. (DOCX)

S2 Table Summary of selected studies. (DOCX)

S1 Checklist PRISMA 2009 checklist. (DOC)

## References

[1] World Health Organization. Every newborn: an action plan to end preventable deaths. 2014.

[2] Blencowe H, Cousens S, Jassir FB, Say L, Chou D, Mathers C et al National, regional, and worldwide estimates of stillbirth rates in 2015, with trends from 2000: a systematic analysis. Lancet Global Health. 2016;4(2):e98–e108. 10.1016/S2214-109X(15)00275-2 26795602

[3] World Health Organization Global strategy for women's, children's and adolescents' health (2016–2030). WHO 2017;2016(9). 10.2471/BLT.16.174714 PMC4850547 27147756

[4] Lawn JE, Blencowe H, Waiswa P, Amouzou A, Mathers C, Hogan D et al Stillbirths: rates, risk factors, and acceleration towards 2030. Lancet. 2016;387(10018):587–603. 10.1016/S0140-6736(15)00837-5 26794078

[5] Mason E, McDougall L, Lawn JE, Gupta A, Claeson M, Pillay Y, et al From evidence to action to deliver a healthy start for the next generation. Lancet. 2014;384(9941):455–67. 10.1016/S0140-6736(14)60750-9 24853599

[6] Pattinson R, Kerber K, Buchmann E, Friberg IK, Belizan M, Lansky S et al Stillbirths: how can health systems deliver for mothers and babies? Lancet. 2011;377(9777):1610–23. 10.1016/S0140-6736(10)62306-9 21496910

[7] Ahmed S, Anastasi E, Laski L. Double burden of tragedy: stillbirth and obstetric fistula. Lancet Global Health. 2016;4(2):e80–e2. 10.1016/S2214-109X(15)00290-9 26823218

[8] Heazell AE, Siassakos D, Blencowe H, Burden C, Bhutta ZA, Cacciatore J et al Stillbirths: economic and psychosocial consequences. Lancet. 2016;387(10018):604–16. 10.1016/S0140-6736(15)00836-3 26794073

[9] Bhutta ZA, Yakoob MY, Lawn JE, Rizvi A, Friberg IK, Weissman E et al Stillbirths: what difference can we make and at what cost? Lancet. 2011;377(9776):1523–38. 10.1016/S0140-6736(10)62269-6 21496906

[10] Bhutta ZA. Counting stillbirths and achieving accountability: A global health priority. PLoS Med. 2017;14(8):e1002364 10.1371/journal.pmed.1002364 28763442 PMC5538629

[11] ten Hoope-Bender P, Stenberg K, Sweeny K. Reductions in stillbirths—more than a triple return on investment. Lancet. 2016;387(10018):e14–e6. 10.1016/S0140-6736(15)01277-5 26794075

[12] Samman E, SDG progress: fragility, crisis and leaving no one behind: report. London: Overseas Developmetn Institute (ODI) 2018.

[13] Frøen JF, Friberg IK, Lawn JE, Bhutta ZA, Pattinson RC, Allanson ER et al Stillbirths: progress and unfinished business. Lancet. 2016;387(10018):574–86. 10.1016/S0140-6736(15)00818-1 26794077

[14] Flenady V, Koopmans L, Middleton P, Frøen JF, Smith GC, Gibbons K et al Major risk factors for stillbirth in high-income countries: a systematic review and meta-analysis. Lancet. 2011;377(9774):1331–40. 10.1016/S0140-6736(10)62233-7 21496916

[15] Aminu M, Unkels R, Mdegela M, Utz B, Adaji S, Van Den Broek N. Causes of and factors associated with stillbirth in low‐and middle‐income countries: a systematic literature review. BJOG. 2014;121:141–53. 10.1111/1471-0528.12995 25236649

[16] Moher D, Shamseer L, Clarke M, Ghersi D, Liberati A, Petticrew M et al Preferred reporting items for systematic review and meta-analysis protocols (PRISMA-P) 2015 statement. Systematic reviews. 2015;4(1):1. 25554246 10.1186/2046-4053-4-1 PMC4320440

[17] Neogi SB, Negandhi P, Chopra S, Mukherjee Das A, Zodpey S, Gupta RK et al Risk Factors for Stillbirth: Findings from a Population-Based Case-Control Study, Haryana, India. Paediatric and Perinat Epidemiology. 2016;30(1):56–66. 10.1111/ppe.12246 26444206

[18] Neogi SB, Sharma J, Negandhi P, Chauhan M, Reddy S, Sethy G. Risk factors for stillbirths: how much can a responsive health system prevent? BMC Pregnancy and Childbirth. 2018;18(1):33 10.1186/s12884-018-1660-1 29347930 PMC5774063

[19] Shabbir S, Zahid M, Qazi A. To detect outcome of pregnancy in advanced maternal age among Pakistani women. Pak Journal of Medical Science. 2014;8(3):709–12.

[20] Owais A, Faruque AS, Das SK, Ahmed S, Rahman S, Stein AD. Maternal and antenatal risk factors for stillbirths and neonatal mortality in rural Bangladesh: a case-control study. PLoS One. 2013;8(11):e80164 10.1371/journal.pone.0080164 24244638 PMC3820579

[21] Lone FW, Qureshi RN, Emanuel F. Maternal anaemia and its impact on perinatal outcome. Tropical Medicine & International Health. 2004;9(4):486–90. 15078267 10.1111/j.1365-3156.2004.01222.x

[22] Jehan I, McClure EM, Salat S, Rizvi S, Pasha O, Harris H et al Stillbirths in an urban community in Pakistan. Am J Obst Gynecol. 2007;197(3):257.e1-8. 10.1016/j.ajog.2007.07.012 PMC2150567 17826410

[23] Bellad MB, Goudar SS, Edlavitch SA, Mahantshetti NS, Naik V, Hemingway-Foday JJ et al Consanguinity, prematurity, birth weight and pregnancy loss: a prospective cohort study at four primary health center areas of Karnataka, India. Journal of Perinatology. 2012;32(6):431–7. 10.1038/jp.2011.115 21852769 PMC13276658

[24] Khan MN, Rahman MM, Shariff AA, Rahman MM, Rahman MS, Rahman MA. Maternal undernutrition and excessive body weight and risk of birth and health outcomes. Archives of Public Health. 2017;75:12 10.1186/s13690-017-0181-0 28174626 PMC5291969

[25] Khanam R, Ahmed S, Creanga AA, Begum N, Koffi AK, Mahmud A et al Antepartum complications and perinatal mortality in rural Bangladesh. BMC Pregnancy and Childbirth. 2017;17(1):81 10.1186/s12884-017-1264-1 28270117 PMC5341426

[26] Lakshmi PVM, Virdi NK, Sharma A, Tripathy JP, Smith KR, Bates MN et al Household air pollution and stillbirths in India: Analysis of the DLHS-II National Survey. Environmental Research. 2013;121:17–22. 10.1016/j.envres.2012.12.004 23375552

[27] Ghimire PR, Agho KE, Renzaho A, Christou A, Nisha MK, Dibley M et al Socio-economic predictors of stillbirths in Nepal (2001–2011). PLoS One. 2017;12(7):e0181332 10.1371/journal.pone.0181332 28704548 PMC5509325

[28] Abir T, Agho KE, Ogbo FA, Stevens GJ, Page A, Hasnat MA et al Predictors of stillbirths in Bangladesh: evidence from the 2004–2014 nation-wide household surveys. Global Health Action. 2017;10(1). 10.1080/16549716.2017.1410048 PMC5757223 29261451

[29] Hossain N, Khan N, Khan NH. Obstetric causes of stillbirth at low socioeconomic settings. Journal of the Pakistan Medical Association. 2009;59(11):744–7. 20361671

[30] Desai G, Anand A, Shah P, Shah S, Dave K, Bhatt H et al Sickle cell disease and pregnancy outcomes: a study of the community-based hospital in a tribal block of Gujarat, India. Journal of Health, Population and Nutrition. 2017;36:1–7. 10.1186/s41043-017-0079-z PMC5251338 28109314

[31] Nisha MK, Alam A, Raynes-Greenow C. Variations in perinatal mortality associated with different polluting fuel types and kitchen location in Bangladesh. International Journal of Occupational and Environmental Health. 2018:24(1–2):47–54. 10.1080/10773525.2018.1507868 30156135 PMC6225514

[32] KC A, Nelin V, Wrammert J, Ewald U, Vitrakoti R, Baral GN, et al Risk factors for antepartum stillbirth: A case-control study in Nepal. BMC Pregnancy and Childbirth. 2015;15(1):146. 26143456 10.1186/s12884-015-0567-3 PMC4491416

[33] KC A, Wrammert J, Ewald U, Clark RB, Gautam J, Baral G et al Incidence of intrapartum stillbirth and associated risk factors in tertiary care setting of Nepal: a case-control study. Reproductive Health. 2016;13(1):103. 27581467 10.1186/s12978-016-0226-9 PMC5007702

[34] Hossain MS, Kypri K, Rahman B, Milton AH. Smokeless tobacco consumption and stillbirth: Population-based case-control study in rural Bangladesh. Drug Alcohol Review. 2018;37(3):414–20. 10.1111/dar.12566 28543690

[35] Newtonraj A, Kaur M, Gupta M, Kumar R. Level, causes, and risk factors of stillbirth: a population-based case control study from Chandigarh, India. BMC Pregnancy and Childbirth. 2017;17:1–9. 10.1186/s12884-016-1183-6 29132325 PMC5684767

[36] Nahar S, Rahman A, Nasreen HE. Factors influencing stillbirth in Bangladesh: A casecontrol study. Paediatric and Perinat Epidemiology. 2013;27(2):158–64. 10.1111/ppe.12026 23374060

[37] International Institute for Population Sciences (IIPS) and ICF. 2017. National Family Health Survey (NFHS-4), 2015–16: India. Mumbai: IIPS.

[38] National Institute of Population Studies (NIPS) [Pakistan] and ICF. 2019. Pakistan Demographic and Health Survey 2017–18. Islamabad, Pakistan, and Rockville, Maryland, USA: NIPS and ICF.

[39] National Institute of Population Research and Training (NIPORT), Mitra and Associates, and ICF International. 2016. Bangladesh Demographic and Health Survey 2014. Dhaka, Bangladesh, and Rockville, Maryland, USA: NIPORT, Mitra and Associates, and ICF International.

[40] Ministry of Health, Nepal; New ERA; and ICF. 2017. Nepal Demographic and Health Survey 2016. Kathmandu, Nepal: Ministry of Health, Nepal.

[41] Xiong T, Mu Y, Liang J, Zhu J, Li X, Li J et al Hypertensive disorders in pregnancy and stillbirth rates: a facility-based study in China. Bulletin of the World Health Organization. 2018; 96(8): 531–539. 10.2471/BLT.18.208447 30104793 PMC6083384

[42] Anggondowati T, EI-Mohandes AA, Qomariyah SN, Kiely M, Ryon JJ, Gipson RF et al Maternal characteristics and obstetrical complications impact neonatal outcomes in Indonesia: a prospective study. BMC pregnancy and childbirth. 2017; 17(1):100 10.1186/s12884-017-1280-1 28351384 PMC5371232

[43] Bayou G, Berhan Y. Perinatal mortality and associated risk factors: a case control study. Ethiopian Journal of Health Science. 2012; 22(3). PMC3511893 23209349

[44] Dahiru T, Aliyu AA. Stillbirth in Nigeria: rates and risk factors based on 2013 Nigeria DHS. Open Access Library Journal. 2016; 3: e2747.

[45] Giang HTN, Bechtold-Dalla Pozza S, Tran HT, Ulrich S. Stillbirth and preterm birth and associated factors in one of the largest cities in central Vietnam. Acta Paediatrica. 2019; 108(4): 630–636. 10.1111/apa.14534 30098081

[46] Akombi BJ, Ghimire PR, Agho KE, Renzaho AM. Stillbirth in the African Great Lakes region: A pooled analysis of Demographic and Health Surveys. PloS one. 2018; 13(8): e0202603 10.1371/journal.pone.0202603 30157230 PMC6114867

[47] Christou A, Dibley MJ, Raynes‐Greenow C. Beyond counting stillbirths to understanding their determinants in low‐and middle‐income countries: a systematic assessment of stillbirth data availability in household surveys. Tropical Medicine & International Health. 2017; 22(3): 294–311. 27992672 10.1111/tmi.12828

