# Supplementary material for: Factors associated with stillbirth in selected countries of South Asia: A systematic review of observational studies
Source: PLoS One. 2020 Sep 16;15(9):e0238938. doi: 10.1371/journal.pone.0238938 (PMC7494090; doi:10.1371/journal.pone.0238938)
Supplement: S1 Table — (DOCX) [file pone.0238938.s001.docx]

S1 Table. Detailed search strategy used in MEDLINE

| SN | Search terms | Results |
| --- | --- | --- |
| 1 | exp Stillbirth/ or exp Fetal Death/ or stillbirth*.mp. or exp Pregnancy Outcome/ | (63083) |
| 2 | exp Fetal Death/ or exp Stillbirth/ or stillborn.mp. | (12819) |
| 3 | exp Pregnancy Outcome/ or exp Stillbirth/ or "fetal death*".mp. | (61752) |
| 4 | exp Pregnancy Outcome/ or "pregnancy outcome*".mp. | (60864) |
| 5 | 1 or 2 or 3 or 4 | (70957) |
| 6 | Risk*.mp. or exp Risk/ or exp Risk Factors/ | (2059600) |
| 7 | factor*.mp. | (3818735) |
| 8 | "risk factor*".mp. or exp Risk Factors/ | (911864) |
| 9 | predictor*.mp. | (295758) |
| 10 | determinant*.mp. | (160438) |
| 11 | etiolog*.mp. | (1639214) |
| 12 | exp Socioeconomic Factors/ or socioeconomic*.mp. | (336726) |
| 13 | sociodemographic*.mp. | (37380) |
| 14 | 6 or 7 or 8 or 9 or 10 or 11 or 12 or 13 | (5916554) |
| 15 | exp Nepal/ or Nepal*.mp. | (8477) |
| 16 | exp India/ or India*.mp. | (125265) |
| 17 | exp Bangladesh/ or Bangladesh*.mp. | (11116) |
| 18 | exp Indian Ocean Islands/ or Maldives*.mp. | (8472) |
| 19 | exp Pakistan/ or Pakistan*.mp. | (18503) |
| 20 | afghan*.mp. or exp Afghanistan/ | (6576) |
| 21 | exp Bhutan/ or Bhutan*.mp. | (642) |
| 22 | exp Sri Lanka/ or Sri lanka*.mp. | (5399) |
| 23 | "Southern Asia*".mp. | (1602) |
| 24 | exp Developing Countries/ or "South Asia*".mp. | (52297) |
| 25 | 15 or 16 or 17 or 18 or 19 or 20 or 21 or 22 or 23 or 24 | (215076) |
| 26 | 5 and 14 and 25 | (1552) |
| 27 | limit 26 to (yr="2000 - 2019" and english) | (1357) |

Note: exp= MeSH headings; mp=keyword

Search Strategy

[Stillbirth* OR stillborn OR “fetal death*” OR “pregnancy outcome*”]

AND

[Risk* OR factor* OR “risk factor*” OR predictor* OR determinant* OR etiolog* OR socio-economic* OR socio-demographic*]

AND

[Nepal* OR India* OR Bangladesh* OR Maldives* OR Pakistan* OR afghan* OR Bhutan* OR Sri lanka* OR “Southern Asia*” OR “South Asia*”]
