# Supplementary material for: Factors associated with stillbirth in selected countries of South Asia: A systematic review of observational studies
Source: PLoS One. 2020 Sep 16;15(9):e0238938. doi: 10.1371/journal.pone.0238938 (PMC7494090; doi:10.1371/journal.pone.0238938)
Supplement: S2 Table — (DOCX) [file pone.0238938.s002.docx]

S 2 Table. Summary of selected studies

| **Author, year** | **Country** | **Study design** | **Sample size** | **Strength and weakness** |
| --- | --- | --- | --- | --- |
| Nisha et al., 2018 | Bangladesh | Cross-sectional | 27237 | Nationally representative sample size; high response rate; high recall period (5 years ) that may be the subject of recall bias; because of retrospective nature of the data, temporal relation cannot be established. |
| Hosain et al., 2018 | Bangladesh | Case-control | 8074 | Exposure data collection without prior knowledge of outcome status and by well‐trained interviewers in face‐to‐face interviews; absence of clinical documentation of pregnancy outcomes and related causes of stillbirth; misreporting, if any, is likely to be non‐differential and therefore would probably lead to underestimation of the association between STC and stillbirth. |
| Khanam et al., 2017 | Bangladesh | Cross-sectional | 6285 | Population-based data and large sample size; self-reported on complication that may raise concern regarding potential misclassification of the reported complications; lack of clinical measures of complications because the survey was conducted in a population that majority of women delivered at home. |
| Abir et al., 2017 | Bangladesh | Cross-sectional | 29094 | High response rate that likely reduces the selection bias; standardized questionnaires used for the pooled analyses of stillbirth; population-based national level sample and findings can be used to formulate country level policies and programs, inclusion of multiple birth that may affect the findings because multiple birth are biologically more likely to die compared to singletons; and studies lacked to include important confounding factors such as health service variables and pregnancy and obstetric factors in the analyses. |
| Khan et al., 2017 | Bangladesh | Cross-sectional | 6548 | Large sample size and findings can be generalized to the wider Bangladeshi population; subject of recall bias because information was retrospectively collected; because of the cross sectional study design, it was unable to establish a causal relationship between the maternal BMI and stillbirth. |
| Nahar et al., 2013 | Bangladesh | Case-control | 22260 | Possible recall bias due to retrospective study design. In addition, the average time interval between childbirth and interview was 4.3 months (range 1–8 months). Classification bias; important factors such as congenital anomalies, fetal growth restriction, utero- placental insufficiency and infections were not included in the study. |
| Owais et al., 2013 | Bangladesh | Cohort | 2317 | Study lacks to control for important confounding factors including undernutrition, possible recall bias due to gap in verbal autopsy and actual death. |
| Neogi et al., 2018 | India | Case-control | 1200 | The use of routine health information system data to identify stillbirths, and ability to include health-system related risk factors with stillbirths. Exclusion of important parameter such as birth weight; and home deliveries and those who never visited the facilities for any post-partum complications were missed out in the study; no verbal autopsies or other medical tests limiting to establish any causation. |
| Newtonraj et al., 2017 | India | Case-control | 18812 | Population based evidence and key findings can be used to drive context-specific programmatic response to reduce stillbirth within its populace; classification of stillbirth is difficult because of retrospective design of the study, and hence program based on classification may be difficult to design. |
| Desai et al., 2017 | India | Cross-sectional | 10035 | Examining the association between SCD and stillbirth that very few studies have done so; exclusion of home deliveries that may reduce the external validity and the findings have limitation for generalization. |
| Neogi et al., 2016 | India | Case-control | 654 | A population-based study with the reporting of immediate cause of stillbirths (medical causes); use of a validated tool to enhance precision of the findings; in-depth interviews of mothers in a familiar setting and the data collection was restricted to 4 months after delivery that may minimize the recall bias; important confounding factors such as birthweight could not be included in this study; quality of reporting stillbirth and its causes is challenging because of different recording procedures in different health facilities. |
| Lakshmi et al., 2013 | India | Cross-sectional | 188917 | Inclusion of both sociodemographic and health variables, large sample size, reporting of early neonatal deaths as stillbirths may result classification bias as the chances of shifting from use of biomass fuel to cleaner fuels are higher than the reverse shift, if any such bias would have occurred, it would pull the prevalence ratio towards the null. |
| Bellad et al., 2012 | India | Cohort | 601 | Kin marriages are common and accepted in the area. If kin marriage were stigmatized, the incidence might have been underestimated and some consanguineous unions might have been misclassified as non- consanguineous. |
| Ghimire et al., 2017 | Nepal | Cross-sectional | 18386 | Pooled analyses with large sample size; high response rate; generalizability of findings to the whole of Nepalese population; inclusion of information five years prior the survey that may be the subject of recall bias; retrospective data and temporal relation cannot be established. |
| KC et al., 2016 | Nepal | Case-control | 25108 | In the absence of periodic national perinatal health survey or a vital registration this study has identified burden of disease and factors associated with stillbirth for intervention; this study is the first study that has examined pregnancy complications on stillbirth; possible underreporting of stillbirth due to lack of medical screening in ANC visits for some of the study population; the use of United States population based reference standards. |
| KC et al., 2015 | Nepal | Case-control | 4567 | First study to examine risk factors for antepartum stillbirth in Nepal, the findings can be useful to reduce the overall stillbirth within its population; lack of placental insufficiency or genetic disorders examination that can have significant association with stillbirth; due to case-control study design, the causal relationship with risk factors cannot be determined. |
| Shabbir et al., 2014 | Pakistan | Cohort | 2401 | The risk of ascertainment bias |
| Hossain et al., 2009 | Pakistan | Cross-sectional | 1011 | Early referrals can decrease the rate of stillbirth from mechanical causes like obstructed labour and ruptured uterus. |
| Jehan et al., 2007 | Pakistan | Cohort | 1280 | Sample from a specific geographic area can be useful to design intervention within its population; high follow- up rate; ultrasound determination of gestational age; relatively small number of stillbirths and the absence of autopsies stand out. |
| Lone et al., 2004 | Pakistan | Cohort | 622 | Hospital based study and the findings cannot be generalized to the whole of country’s population. Selection bias due to the inclusion of sample only from tertiary care hospital as the burden of anaemia can be expected to be much higher outside the hospital setting. |
